# Effects of Supplementing Rumen-Protected Lysine and Methionine on Apparent Digestibility, Rumen Fermentation Parameters, and Microbial Profiles in Lactating Dairy Cows Under Different Environmental Conditions

**DOI:** 10.3390/ani15233439

**Published:** 2025-11-28

**Authors:** Ruoran Tao, Ke Wang, Xing Han, Xu Tang, Dian Wang, Yuhang Ding, Yuhong Ma, Maocheng Jiang, Sijia Liu, Yinghao Huang, Caiyun Fan, Zhao Zhuo, Jianbo Cheng

**Affiliations:** 1 College of Animal Science and Technology, Anhui Agricultural University, Hefei 230036, China; 15956548294@163.com (R.T.);; 2 National Center of Technology Innovation for Dairy, Hohhot 010010, China; 3 Inner Mongolia Youran Dairy Group Limited, Hohhot 010010, China; 4 Shanghai Menon Biotechnology Co., Ltd., Shanghai 201800, China

**Keywords:** rumen-protected methionine and lysine, apparent digestibility, rumen fermentation, microorganisms, heat stress

## Abstract

This study investigated the effects of supplementing rumen-protected lysine (RPL; 60 g/d) and methionine (RPM; 30 g/d) on nutrient digestibility, rumen fermentation, and microbial composition in 30 early-lactation Holstein dairy cows under both heat stress (HS) and non-heat stress (NHS) conditions. Cows were paired based on parity, milk yield, and body weight, and then assigned to one of two dietary treatments: the control group (CON), receiving the basal diet only, or the RPLM group, which received the basal diet supplemented with 60 g/d of RPL and 30 g/d of RPM (*n* = 15 per group). All animals underwent a transition from HS to NHS conditions during the experimental period, allowing for within-animal comparison across thermal environments. Results demonstrated that RPLM supplementation significantly improved the apparent digestibility of dry matter (DM), crude protein (CP), and neutral detergent fiber (NDF) (*p* < 0.05), with a significant treatment × environment interaction observed for DM digestibility (*p* < 0.01). Under HS conditions, RPLM reduced ruminal NH_3_-N concentration (*p* < 0.05) and increased total volatile fatty acids (TVFA), acetate, and butyrate levels (*p* < 0.01), suggesting enhanced nitrogen utilization and energy metabolism. No significant effects were observed on ruminal pH or the acetate-to-propionate ratio. 16S rRNA sequencing revealed that RPLM supplementation increased the relative abundance of fiber-degrading and butyrate-producing taxa, including *NK4A214_group*, *Christensenellaceae_R-7_group*, *norank_f__Muribaculaceae*, and *norank_f__F082*. These microbial taxa exhibited strong positive correlations with DM and CP digestibility (*p* < 0.001). LEfSe analysis further confirmed their status as key microbial biomarkers in the RPLM-NHS group. Collectively, concurrent supplementation of RPL and RPM modulates the ruminal microbiota, alleviates HS-induced reductions in digestive efficiency, and enhances overall nutrient utilization in dairy cows.

## Simple Summary

Thirty lactating Holstein dairy cows were randomly assigned in a paired design to evaluate the effects of a basal diet (CON) versus the same basal diet supplemented with 60 g/d rumen-protected lysine and 30 g/d of rumen-protected methionine (RPLM) under both heat stress (HS) and non-heat stress (NHS) conditions. Supplementation with RPLM significantly improved the apparent digestibility of dry matter, crude protein, and neutral detergent fiber. During the HS period, rumen ammonia nitrogen concentration decreased, while concentrations of TVFA, acetate, and butyrate increased markedly. 16S rRNA sequencing analysis revealed that RPLM supplementation enriched butyrate-producing bacterial taxa, particularly the *NK4A214_group*, which showed a positive correlation with nutrient digestibility. These findings indicate that RPLM enhances rumen microbial structure and nutrient utilization efficiency, thereby mitigating heat stress-induced digestive impairments.

## 1. Introduction

Heat stress is a prevalent environmental stressor in dairy cattle farming that significantly compromises the production performance, health status, and economic returns of dairy operations. Under high ambient temperatures, dairy cows exhibit a marked reduction in feed intake, leading to insufficient energy and nutrient intake. This deficiency subsequently impairs milk yield, milk composition, and reproductive efficiency. Furthermore, heat stress can induce immune dysfunction and oxidative stress, thereby increasing disease susceptibility and reducing animal welfare and productive longevity. Therefore, mitigating the adverse effects of heat stress has become a critical challenge for the modern dairy industry. In recent years, nutritional intervention has emerged as an effective strategy to alleviate the impacts of heat stress. As fundamental building blocks for protein synthesis, amino acids play a crucial role in maintaining normal physiological functions and optimizing production performance in dairy cows. Methionine and lysine are two key limiting amino acids with pivotal roles in dairy cattle nutrition and metabolism. Methionine is classified as an essential amino acid and is commonly recognized as the first-limiting nutrient in diets based on soybean meal [1,2]. Previous studies have demonstrated that direct supplementation of high-dose free amino acids can theoretically meet the nutritional requirements of dairy cows; however, this approach may induce toxicological responses in some cases. Furthermore, while free amino acids administered directly may not effectively influence those metabolized in the small intestine, the supply of amino [3] acids derived from microbial protein and rumen-bypass substances can be modulated [4]. Currently, rumen-protected amino acids, delivered through coating or microencapsulation technologies, effectively resist ruminal degradation and reach the small intestine in a more stable and predictable manner. This results in significantly improved nitrogen utilization efficiency, enhanced milk protein synthesis, and better overall production performance. In contrast, free amino acids are subject to extensive ruminal degradation and unpredictable metabolic responses, making them unsuitable for direct inclusion in dairy cow diets [4,5,6].

Methionine functions as a key amino acid involved in various biological processes, including DNA methylation, translational regulation, the synthesis of additional biomolecules, and maintaining antioxidant homeostasis [7,8]. Research indicates that supplementing dairy cow diets with rumen-protected methionine (RPM) can lead to increased milk protein levels during both prepartum and postpartum periods [9,10], even though overall milk yield and protein output do not consistently rise. Lysine exerts not only a profound influence on milk protein synthesis but also contributes substantially to enhancing the overall productivity of dairy cattle. As a key limiting amino acid in Ruminants [1], it performs an indispensable function in the mammary gland during protein biosynthesis [11]. Administration of RPL enhances its availability in the duodenum and improves the uptake and utilization efficiency of other essential amino acids (EAA) by mammary tissues [12]. Nevertheless, some studies have reported no significant alterations in intestinal amino acid flow or nutrient digestibility following RPL supplementation [13,14]. Currently, numerous studies have investigated the effects of supplementing ruminant diets with RPL and RPM on production performance. However, emerging evidence suggests that despite encapsulation, these supplemented amino acids may still influence the composition and activity of the ruminal microbial community [15,16,17]. Emerging evidence demonstrates that heat stress activates the hypothalamic-pituitary-adrenal (HPA) axis, leading to a 30–50% increase in plasma corticosterone levels. This hormonal shift upregulates muscle F-box atrophy-related genes and suppresses the mTOR-S6K1 signaling pathway, triggering the efflux of essential amino acids—such as lysine and arginine—from skeletal muscle. These amino acids serve as carbon skeletons for hepatic gluconeogenesis [18]. Additionally, heat stress is associated with reduced feed intake, which directly alters endocrine status and metabolic flux, including enhanced catabolism of branched-chain amino acids (BCAAs) to meet energy demands [19,20]. Concurrently, heat stress disrupts gut microbial homeostasis, potentially increasing microbial competition for host-derived amino acids [21]. Together, these mechanisms highlight a systemic repartitioning and reutilization of amino acids during heat stress, reflecting a coordinated physiological adaptation to metabolic challenge.

Therefore, this study aims to investigate whether co-supplementation of RPL and RPM can alleviate heat stress in dairy cows, and to further examine how differing environmental conditions (heat stress vs. non-heat stress) influence rumen fermentation parameters and microbial composition. In contrast to previous studies that have largely examined these factors in isolation, this research specifically evaluates the interaction between RPAA supplementation and environmental conditions to elucidate the integrated effects of amino acid nutrition, thermal environment, and ruminal function. Additionally, we explore how heat stress-induced systemic amino acid reutilization affects the rumen microbiota. A comprehensive analysis of these mechanisms will enhance understanding of the functional roles of RPL and RPM in dairy production. The findings are expected to provide robust scientific evidence and novel insights for mitigating heat stress, advancing precision nutrition, and refining amino acid balancing strategies in dairy farming.

## 2. Materials and Methods

### 2.1. Experiment Design, Diets and Environment

Thirty healthy Holstein dairy cows (milk yield = 38.05 ± 5.35 kg; average parity = 1.89 ± 1) were randomly assigned to one of two dietary treatments: a total mixed ration (TMR) supplemented with 60 g/d of rumen-protected lysine (RPL) and 30 g/d of rumen-protected methionine (RPM) (RPL and RPM sourced from Shanghai Menon Biotechnology Co., Ltd. (Shanghai, China); RPL: L-lysine hydrochloride ≥ 60%, rumen escape rate ≥ 90%, small intestine release rate ≥ 90%; RPM: DL-methionine ≥ 75%, rumen escape rate ≥ 90%, small intestine release rate ≥ 90%) (RPLM group), or an unsupplemented control TMR (CON group). Within each treatment group, all cows transitioned from a heat stress (HS) period to a non-heat stress (NHS) period. To minimize the influence of lactation stage, all cows were maintained in early lactation throughout both experimental phases. The study was conducted in two distinct periods: the HS period (35d) and the NHS period (30d), and these two periods were consecutive. During the HS period, cows were exposed to heat stress conditions (temperature = 26.2 ± 3.5 °C; relative humidity = 85.1 ± 8.5%; THI = 76.2 ± 2), calculated as THI = T°C dry bulb − [0.55 − (0.55 × RH/100)] × (T°C dry bulb − 58) [22]. Environmental temperature and humidity in the experimental barn were monitored three times daily at 06:00, 12:00, and 22:00 using a data logging system. After the heat stress period ended, a 7-day transition period was implemented during which the thermal comfort index (THI) remained below the heat stress threshold and the ventilation system was adjusted daily to allow the cows to gradually adapt, after which the cows entered the non-stress lactation period. During the NHS period, cows were housed under thermoneutral conditions (temperature = 18.1 ± 3.2 °C; relative humidity = 78.3 ± 6.9%; THI = 63.3 ± 2). All cows followed the same sequential transition from the HS to the NHS period without crossover in treatment order; however, individual animal effects were balanced through within-subject randomization. All cows were fed a total mixed ration (TMR) and had continuous access to water.

Throughout the entire experiment, all cows received the same basal TMR, formulated to meet the nutritional requirements for dairy cattle (NRC, 2001), with feeding times at 04:00, 12:00, and 20:00 daily. Supplements of 60 g/d rumen-protected lysine and 30 g/d rumen-protected methionine were administered based on farm-specific TMR composition and in accordance with recommendations from the literature [1,23], maintaining a dietary lysine-to-methionine ratio of 3:1. Dietary composition and nutrient levels are presented in Table 1. All diets were freshly prepared daily and offered as TMR, with feed and water available ad libitum.

### 2.2. Data Collection and Sampling Procedure

During the experiment, three samples of total mixed ration (TMR) were collected daily and thoroughly homogenized to ensure uniformity prior to feed composition analysis. The samples were dried at 65 °C for 48 h until a constant weight was reached. After drying, the TMR samples were ground using a 1-mm sieve (KRT-34; Kunjie, Beijing, China), and the dry matter (DM) content was determined following AOAC Method 950.15. Nitrogen content was analyzed using AOAC Method 984.1 [24], and crude protein (CP) was calculated by multiplying nitrogen content by 6.25. Neutral detergent fiber (NDF) and acid detergent fiber (ADF) contents were quantified in accordance with the procedure described by Van Soest et al. [25].

Fecal samples were collected from each selected dairy cow over two consecutive days during both HS and NHS periods. On each day, three samples were collected per cow at predetermined time points (03:00, 13:00, and 18:00). All daily samples from the same cow were thoroughly mixed to form a composite sample, and approximately 200 g of the mixture was retained for subsequent analysis. The collected samples were then dried in a forced-air oven set at 65 °C until they reached a stable weight, followed by grinding to pass through a 1-mm sieve (model KRT-34; Kunjie, Beijing, China). The levels of dry matter (DM), neutral detergent fiber (NDF), acid detergent fiber (ADF), crude protein (CP), and ether extract (EE) in the fecal samples were assessed using the AOAC method as referenced earlier. Acid-insoluble ash (AIA) served as an internal marker to estimate the apparent digestibility of nutrients in the diet. The analyses of total mixed ration (TMR), leftover feed, and fecal materials were conducted following the protocol established by Van Kuren and Yang [26]. The equation used to calculate apparent nutrient digestibility is presented below:

Apparent digestibility of nutrients (%) = [1 − (Ad × Nf)/(Af × Nd)] × 100

where Ad and Af represent the AIA content in feed and feces (g/kg), respectively; Nf and Nd represent the content of the corresponding nutrient in feces and feed (g/kg), respectively.

Rumen fluid samples were collected from six dairy cows randomly selected from the CON and RPLM groups prior to the end of both HS and NHS phases. Sampling was conducted before morning feeding using an oral rumen tube connected to a vacuum pump to obtain representative rumen fluid samples. To reduce contamination by saliva, the first 50 mL of rumen fluid was discarded, and approximately 150 mL of the following sample was collected. Immediately after collection, the pH level of each sample was recorded. Afterwards, portions of each sample were placed into centrifuge tubes and kept at −20 °C for future analysis of volatile fatty acids (VFA), ammonia nitrogen (NH_3_-N), and microbial protein (MCP) levels. Furthermore, filtered rumen fluid was portioned into cryovials (two per animal) and kept in liquid nitrogen for analysis of microbial community structure.

The concentration of volatile fatty acids (VFA) was analyzed using a GC-2010 gas chromatograph (Shimadzu, Kyoto, Japan). The procedure was as follows: 20 mL of rumen fluid sample was centrifuged at 13,000 rpm and 4 °C for 15 min. One milliliter of the supernatant was then transferred into a 1.5 mL centrifuge tube, followed by the addition of 0.2 mL of 25% orthophosphoric acid solution containing the internal standard 2EB. The mixture was vortexed thoroughly and incubated on ice for at least 30 min. Afterward, the sample was centrifuged again at 15,000 rpm for 15 min. Two milliliters of the resulting supernatant were filtered through a 0.22 μm aqueous membrane filter prior to HPLC analysis. Chromatographic conditions were set as follows: column, HP-INNOWAX (1909IN-133); injector temperature, 200 °C; detector temperature, 220 °C; oven temperature program, initial temperature held at 80 °C for 1 min, ramped at 15 °C/min to 170 °C, and maintained for 15 min; carrier gas, high-purity nitrogen at a pressure of 100 kPa, with a total flow rate of 63.8 mL/min, column flow rate of 1.19 mL/min, split ratio of 50, purge flow rate of 3 mL/min, and circulation flow rate of 30 mL/min; detector gas flow rates, hydrogen at 40 mL/min and air at 400 mL/min; injection volume, 2 μL.

The concentration of ammonia nitrogen (NH_3_-N) was determined using the phenol-sodium hypochlorite method [27], while microbial protein (MCP) was quantified using the Coomassie Brilliant Blue G250 method. The frozen rumen fluid samples were sent to Shanghai Majorbio Bio-pharm Technology Co., Ltd. (Shanghai, China). for 16S rRNA sequencing analysis of the rumen bacterial community.

### 2.3. Rumen Microorganism DNA Extraction and PCR Amplification

Total microbial genomic DNA was extracted from rumen liquid samples using the E.Z.N.A.^®^ soil DNA Kit (Omega Bio-tek, Norcross, GA, USA) according to manufacturer’s instructions. The quality and concentration of DNA were determined by 1.0% agarose gel electrophoresis and a NanoDrop2000 spectrophotometer (Thermo Scientific, Waltham, MA, USA) and kept at −80 °C prior to further use. The hypervariable region V3–V4 of the bacterial 16S rRNA gene were amplified with primer pairs 338F (5′-ACTCCTACGGGAGGCAGCAG-3′) and 806R(5′-GGACTACHVGGGTWTCTAAT-3′) [28] by T100 Thermal Cycler PCR thermocycler (BIO-RAD, Hercules, CA, USA). The PCR reaction mixture including 4 μL 5 × Fast Pfu buffer, 2 μL 2.5 mM dNTPs, 0.8 μL each primer (5 μM), 0.4 μL Fast Pfu polymerase, 10 ng of template DNA, and ddH_2_O to a final volume of 20 µL. PCR amplification cycling conditions were as follows: initial denaturation at 95 °C for 3 min, followed by 27 cycles of denaturing at 95 °C for 30 s, annealing at 55 °C for 30 s and extension at 72 °C for 45 s, and single extension at 72 °C for 10 min, and end at 4 °C. The PCR product was extracted from 2% agarose gel and purified using the PCR Clean-Up Kit (YuHua, Shanghai, China) according to manufacturer’s instructions and quantified using Qubit 4.0 (Thermo Fisher Scientific, USA). A negative control (CK) was included in the PCR amplification process, in which sterile water was used instead of template DNA. This control was subjected to the same reagents and thermal cycling conditions as the experimental samples. The absence of amplification bands in the gel electrophoresis image of the negative control confirms that the PCR reagents were free from contamination, thereby minimizing the risk of false positive results and supporting the validity and reliability of the experimental outcomes.

### 2.4. Analysis of Sequencing Data

Purified amplicons were pooled in equimolar amounts and paired-end sequenced on an Illumina Nextseq2000 platform (Illumina, San Diego, CA, USA) according to the standard protocols by Majorbio Bio-Pharm Technology Co., Ltd. (Shanghai, China). The raw sequencing reads were deposited into the NCBI Sequence Read Archive (SRA) database (Accession Number SRP604849). Reviewer links for PRJNA1299118: Please see “https://www.ncbi.nlm.nih.gov/sra/?term=PRJNA1299118, accessed on 19 July 2025” The datasets used and/or analyzed during the current study are available from the corresponding author on reasonable request. https://www.ncbi.nlm.nih.gov/sra/?term=PRJNA1299118, accessed on 19 July 2025. To minimize the influence of sequencing depth on downstream analyses of alpha and beta diversity, all samples were rarefied to an equal sequencing depth of 32,558 reads.

### 2.5. Statistical Analyses

All data were analyzed using SAS software (version 9.2; SAS Institute Inc., Cary, NC, USA), with dairy cows serving as the experimental units. The apparent digestibility of nutrients and rumen fermentation products were evaluated using the PROC MIXED procedure in SAS. The statistical models for these variables included fixed effects of treatment, environment, and their interaction (treatment × environment), with cows nested within treatment groups treated as a random effect. Degrees of freedom were estimated using the Kenward-Roger approximation method. Differences between the treatment and control groups were considered statistically significant at *p* < 0.05 and highly significant at *p* < 0.01.

At the same time, an independent-sample *t*-test was conducted to compare differences between the CON and RPLM groups under identical environmental conditions. Results were reported as mean ± standard error (mean ± SE). Statistical significance was defined using two thresholds: *p* < 0.05 was considered statistically significant, whereas *p* < 0.01 was regarded as indicating highly significant differences between experimental groups. Principal coordinate analysis (PCoA) was performed to evaluate β-diversity based on the weighted UniFrac distance [29]. Furthermore, Spearman correlation coefficients were calculated to examine the associations between rumen microbial communities and fermentation parameters [30]. One-way ANOVA was used to identify microbial taxa with significantly different abundances across groups. The *p*-values from the ANOVA tests were adjusted for multiple comparisons using the false discovery rate (FDR) method, and differences were considered statistically significant at FDR-adjusted *p* < 0.05. Subsequently, linear discriminant analysis (LDA) was applied to estimate the effect size of each species’ abundance in driving group differences.

## 3. Results

### 3.1. Environmental Temperature Indicators

**Figure 1:**
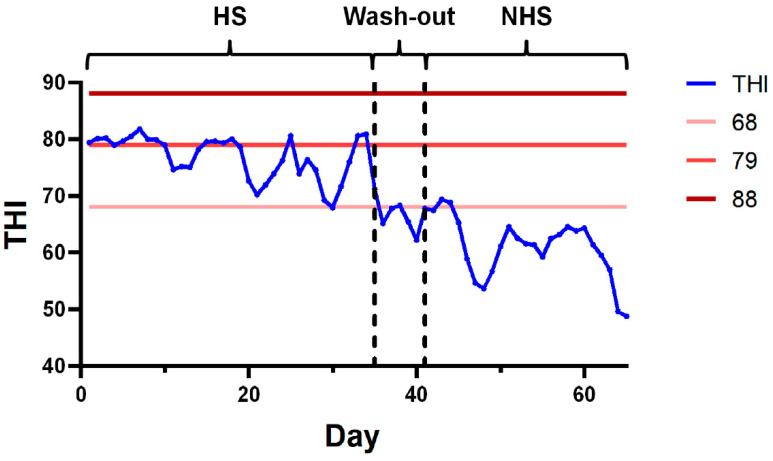
Temperature and Humidity Profiles in the Experimental Cattle Housing Facility.

THI within the cattle housing facility was monitored at 06:00, 12:00, and 22:00 daily, and the daily average THI values were subsequently calculated (Figure 1). During the observation period, dairy cows experienced heat stress conditions (THI ≥ 68) for a total of 35 days. Following a 7-day transition phase, the majority of cows had adapted and were no longer under heat stress (THI < 68).

### 3.2. Effects of Supplementing RPL and RPM on Apparent Digestibility in Dairy Cows

**Figure 2:**
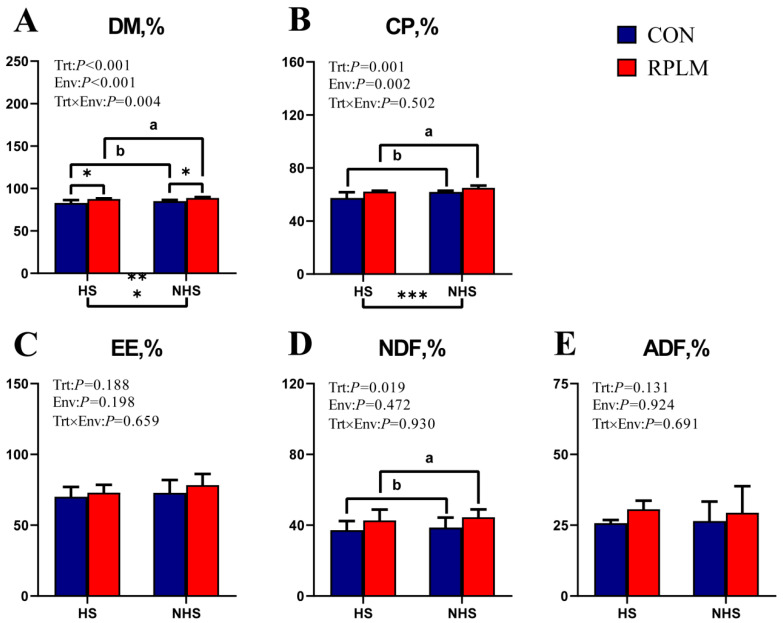
The effects of adding RPL and RPM in different environmental conditions on the apparent digestibility of dairy cows were investigated. Multivariate linear regression analysis was used to assess the relationships between variables and their potential interactions. *t*-tests were conducted on the indicators with interaction effects to compare the differences between the two groups under the same environmental conditions. Significant associations were annotated with asterisks: * for 0.01 < *p* ≤ 0.05, ** for 0.001 < *p* ≤ 0.01, *** for *p* ≤ 0.001. Different letters indicated significant differences among the treatments (*p* < 0.05). The data are presented as mean ± SE (*n* = 12–14). (**A**) Dry matter apparent digestibility; (**B**) Crude protein apparent digestibility; (**C**) Crude fat apparent digestibility; (**D**) Crude cellulose apparent digestibility; (**E**) Acid detergent fiber apparent digestibility.

As shown in Figure 2, a highly significant interaction between treatment and environment was observed for the apparent digestibility of DM in the RPLM group (*p* < 0.01). Furthermore, under all tested environmental conditions, the apparent DM digestibility in the RPLM group was significantly higher than that in the CON group. In contrast, no significant interaction effects of treatment and environment were detected for CP, EE, NDF, or ADF. However, the apparent digestibility of both CP and NDF was significantly greater in the RPLM group compared to the CON group, indicating a significant treatment effect (*p* < 0.05). Notably, the enhancement in CP digestibility was significantly influenced by environmental factors (*p* = 0.002).

### 3.3. Effects of Supplementing Rumen-Protected Lysine and Methionine on Rumen Fermentation Parameters in Dairy Cows

**Figure 3:**
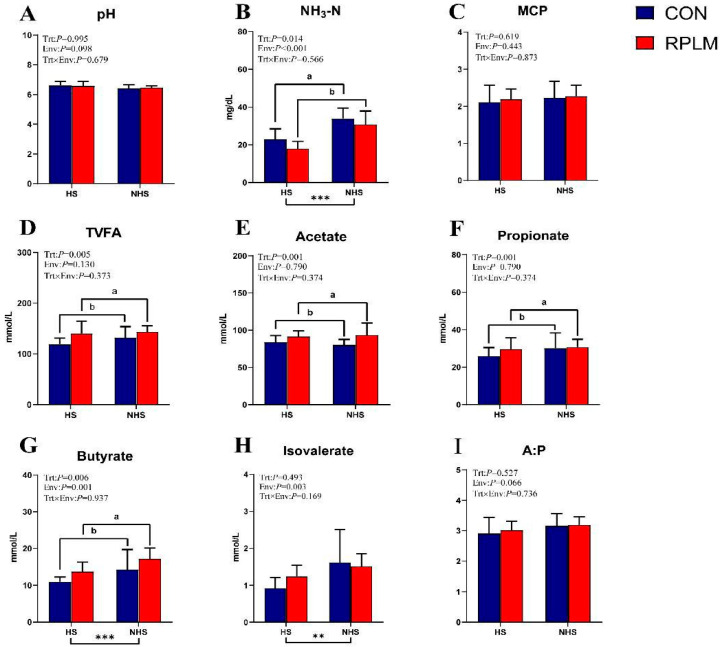
The effects of adding RPL and RPM in different environmental conditions on rumen fermentation parameters of dairy cows were investigated. Multivariate linear regression analysis was used to assess the relationships between variables and their potential interactions. *t*-tests were conducted on the indicators with interaction effects to compare the differences between the two groups under the same environmental conditions. Significant associations were annotated with asterisks: * for 0.01 < *p* ≤ 0.05, ** for 0.001 < *p* ≤ 0.01, *** for *p* ≤ 0.001. Different letters indicated significant differences among the treatments (*p* < 0.05). The data are presented as mean ± SE (*n* = 8) (**A**) pH; (**B**) NH3-N; (**C**) MCP; (**D**) total volatile fatty acids; (**E**) acetate; (**F**) propionate; (**G**) butyrate; (**H**) isovalerate; (**I**) ratio of acetate to propionate (A:P).

An interaction analysis of rumen fermentation parameters was conducted (Figure 3). The results revealed no significant differences in ruminal pH values between the CON and PLM groups across varying environmental conditions (*p* > 0.05). In the analysis of NH_3_-N, supplementation with RPL and RPM showed a significant treatment effect (*p* < 0.05), along with a highly significant environmental effect (*p* < 0.01). Compared to the CON group, the RPLM group exhibited a highly significant increase in TVFA, acetate, propionate, and butyrate concentrations (*p* < 0.01). Notably, butyrate concentration was also significantly influenced by environmental factors (*p* < 0.01). Similarly, the increase in isovalerate was significantly affected by environmental conditions (*p* < 0.01). Furthermore, no significant changes in the A:P ratio were observed across different environmental conditions, indicating a consistent rumen fermentation pattern in dairy cows. This stability is likely attributable to the uniformity of the dietary structure throughout the experiment. As the diet composition for early lactating cows remained unchanged during the entire experimental period, the constant ratio of fiber to starch may have supported a stable microbial fermentation environment in the rumen. None of the aforementioned rumen fermentation parameters demonstrated a significant interaction effect between treatment and environmental factors.

### 3.4. Effects of Supplementing Rumen-Protected Lysine and Methionine on Rumen Microbial Communities in Dairy Cows

**Figure 4:**
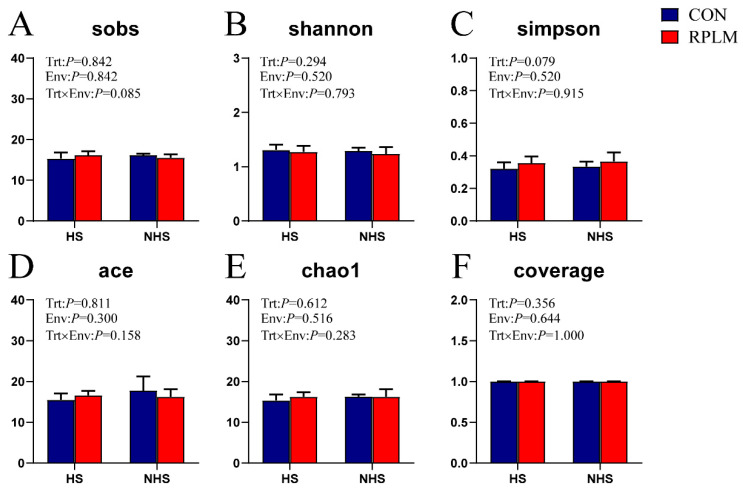
Graphical summary of the Alpha-Diversity Index of Rumen Bacterial Communities. CON-HS = The CON group was fed with the basal diet during the heat-stress period. RPLM-HS = The RPLM group received an additional supplementation of 60 g/d RPL and 30 g/d RPM on the basal diet during the heat-stress period. CON-NHS = The CON group was fed with the basal diet during the non-heat-stress period. RPLM-NHS = The RPLM group received an additional supplementation of 60 g/d RPL and 30 g/d RPM on the basal diet during the non-heat-stress period. Data are expressed as mean ± SE, *n* = 6. (**A**) ACE index; (**B**) Chao 1 index; (**C**) Coverage index; (**D**) Shannon index; (**E**) Simpson index; (**F**) Sobs index.

Figure 4 shows no significant differences in the α-diversity indices of rumen bacterial communities between the CON and RPLM groups across different environmental conditions (*p* > 0.05). The relatively high Shannon index values and low Simpson index values indicate a uniform species distribution within the microbial community. All samples exhibited sequencing coverage exceeding 0.99, confirming high species richness and diversity in the rumen bacterial population. These results demonstrate that the sequencing depth was sufficient to reliably capture the structural composition of the microbiota.

**Figure 5:**
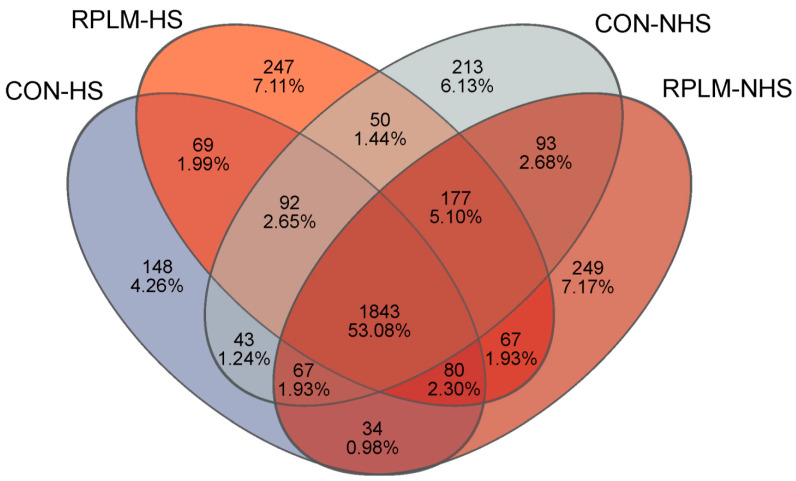
Venn diagram for the distribution of bacterial OTUs in the rumen.

In order to study the species composition of each sample, all samples were clustered into operational taxonomic units (OTUs) and subjected to taxonomic annotation. As shown in Figure 5, there were obvious differences in the OTU distribution between the CON group and the RPLM group during the HS and NHS periods. Specifically, there were 1843 OTUs shared by the four groups, 2084 OTUs shared by the CON-HS and RPLM-HS groups, and 2180 OTUs shared by the CON-NHS and RPLM-NHS groups. This indicates a high degree of similarity in their species composition. In addition, CON-HS, RPLM-HS, CON-NHS, and RPLM-NHS contained 148, 247, 213, and 249 unique OTUs respectively. This shows that the number of unique OTUs in the CON group during the HS period differed significantly from that in the CON group during the NHS period and that in the RPLM group throughout the entire experimental period.

**Figure 6:**
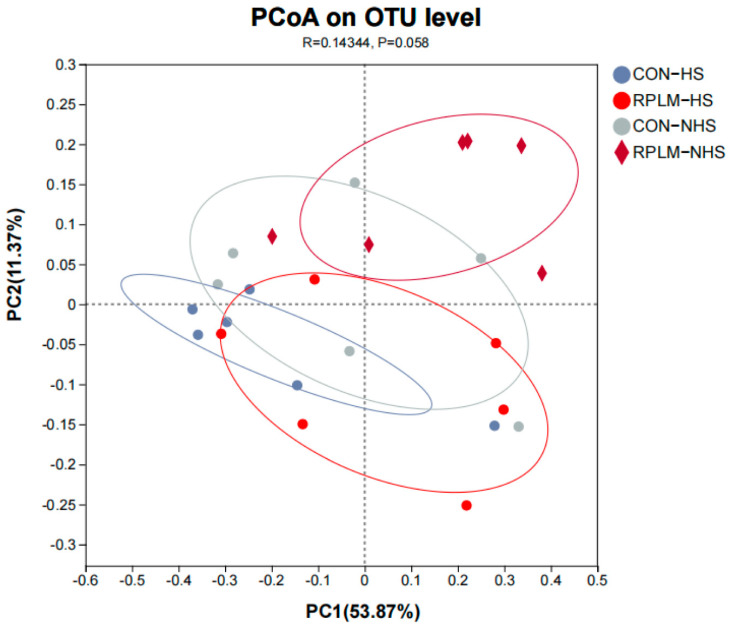
Principal coordinate analysis (PCOA) of rumen microbiota based on Bray–Curtis dissimilarity of OTU composition. *n* = 6.

To visually display the differences in microbial community structure, we employed principal coordinates analysis (PCoA). As shown in Figure 6, in the two principal coordinates analyses, the clusters of CON-HS, RPLM-HS, and CON-NHS were not clearly separated, but the clusters of RPLM-NHS were significantly separated from those of CON-HS and RPLM-HS. Further analysis revealed that the first two principal components, namely principal component 1 (PC1) and principal component 2 (PC2), explained 53.87% and 11.37% of the total variation, respectively. In conclusion, based on the principal coordinates analysis, it can be inferred that during the HS period, the CON group and the RPLM group exhibited similar community composition and species abundance, while during the NHS period, there were significant differences between the CON group and the RPLM group, indicating that the addition of RPM and RPL had a significant impact. Meanwhile, the community composition and species abundance of RPLM-NHS differed greatly from those of CON-HS, suggesting that this phenomenon might be jointly produced by the interaction between the treatment and the environment.

**Figure 7:**
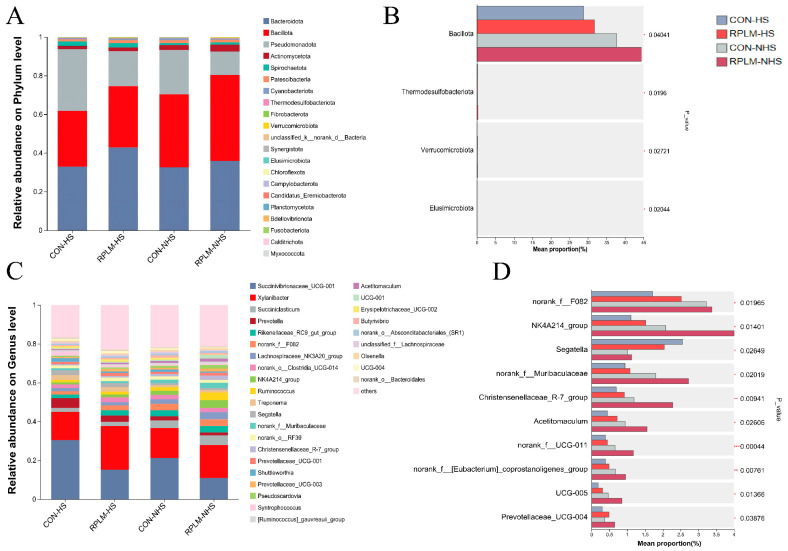
Species composition analysis identified the top 50 bacterial phyla and genera based on relative abundance at both the phylum and genus levels. To evaluate inter-group differences in microbial community structure, one-way ANOVA was performed to assess significant variations between the CON and RPLM groups at these taxonomic levels. (**A**) Composition of abundances of rumen microbiota at the phylum level in the CON-HS, RPLM-HS, CON-NHS and RPLM-NHS groups; (**B**) Comparison of abundances of rumen microbiota at the phylum level in the CON-HS, RPLM-HS, CON-NHS and RPLM-NHS groups; (**C**) Composition of abundances of rumen microbiota at the genus level in the CON-HS, RPLM-HS, CON-NHS and RPLM-NHS groups; (**D**) Comparison of abundances of rumen microbiota at the genus level in the CON-HS, RPLM-HS, CON-NHS and RPLM-NHS groups.Statistically significant associations were marked with asterisks: * for 0.01 < *p* ≤ 0.05, ** for 0.001 < *p* ≤ 0.01, and *** for *p* ≤ 0.001.

Figure 7A,C illustrate the variations in bacterial community composition at the phylum and genus levels in the control group (CON group) and the RPLM group under different environmental conditions (HS/NHS). Based on species annotation results, the top ten most abundant phyla in each group were selected to construct relative abundance bar charts. Figure 4B,D highlight the phyla and genera that exhibited significant differences among the four groups.

At the phylum level, *Bacteroidota* and *Bacillota* were consistently dominant bacterial groups across both experimental groups under different environmental conditions. The relative abundances of *Bacteroidota* and *Bacillota* in RPLM-HS and RRPLM-NHS were significantly higher compared to the CON group (CON-HS and CON-NHS). Conversely, the relative abundance of *Actinomycetota* was markedly lower in the RPLM group than in the CON group. One-way ANOVA revealed statistically significant differences in the abundances of several bacterial phyla—including *Bacillota*, *Thermodesulfobacteriota*, *Verrucomicrobiota*, and *Elusimicrobiota*—across the groups under varying conditions. Notably, both the CON and RPLM groups exhibited similar temporal trends in rumen microbial composition during the transition from HS to NHS, suggesting a consistent physiological response to dietary or environmental shifts. Under varying environmental conditions, the rumen fluid of both the CON group and the RPPLM group showed the highest relative abundances of *Succinivibrionaceae_UCG-001* and *Xylanibacter*, indicating their dominance within the microbial community. At the genus level, the rumen microbiota of lactating dairy cows demonstrated high diversity. Significant differences were observed in the relative abundances of *norank_f__F082*, *NK4A214_group*, *Segatella*, *norank_f__Muribaculaceae*, *Acetitomaculum*, *UCG-005*, and *Prevotellaceae_UCG-004* among the four groups (*p* < 0.05), while extremely significant differences were found for *Christensenellaceae_R-7_group*, *norank_f__UCG-011*, and *norank_f__[Eubacterium]_coprostanoligenes_group* (*p* < 0.01).

**Figure 8:**
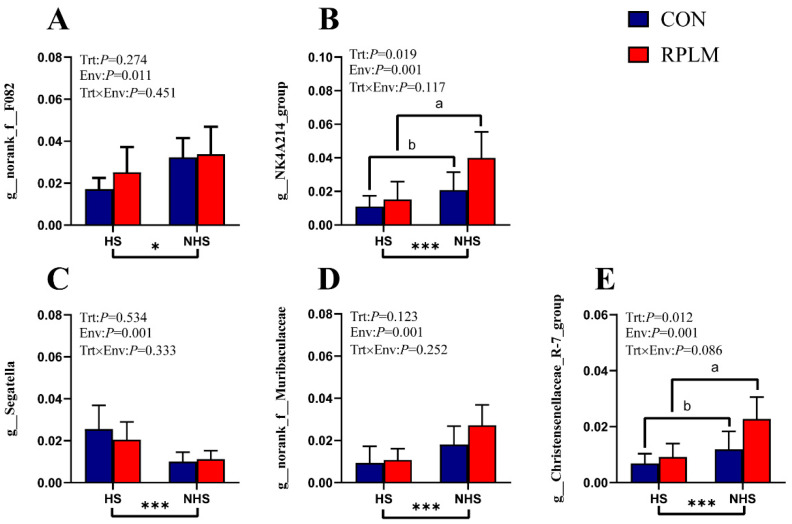
Four bacterial genera with relative abundances exceeding 0.5% were selected for further analysis. Multivariate linear regression analysis was conducted to evaluate the relationships among variables and identify potential interaction effects. For indicators showing significant interaction effects, independent-sample *t*-tests were performed to compare differences between the two groups under identical environmental conditions. Statistically significant associations were marked with asterisks: * for 0.01 < *p* ≤ 0.05, ** for 0.001 < *p* ≤ 0.01, and *** for *p* ≤ 0.001. Different lowercase letters denote significant differences among treatments (*p* < 0.05). Data are expressed as mean ± SE (*n* = 6). (**A**) *g_norank_f__F082*; (**B**) *g_NK4A214_group*; (**C**) *g_Segatella*; (**D**) *g_norank_f__Muribaculaceae*; (**E**) *g_Christensenellaceae_R-7_group*.

Bacterial genera with a relative abundance exceeding 0.5% among the differential taxa were selected for interaction analysis (Figure 8). *t*-tests were also conducted to compare the CON and RPLM groups under identical environmental conditions. The results indicated that, apart from *g_norank_f__F082*, which exhibited a significant environmental effect, all other selected differential genera demonstrated highly significant environmental effects (*p* = 0.001). Furthermore, *g_NK4A214_group* and *g_Christensenellaceae_R-7_group* showed significant treatment effects (main effects) (*p* < 0.05). No significant interaction effects between treatment and environmental factors were observed for any of the selected differential genera.

**Figure 9:**
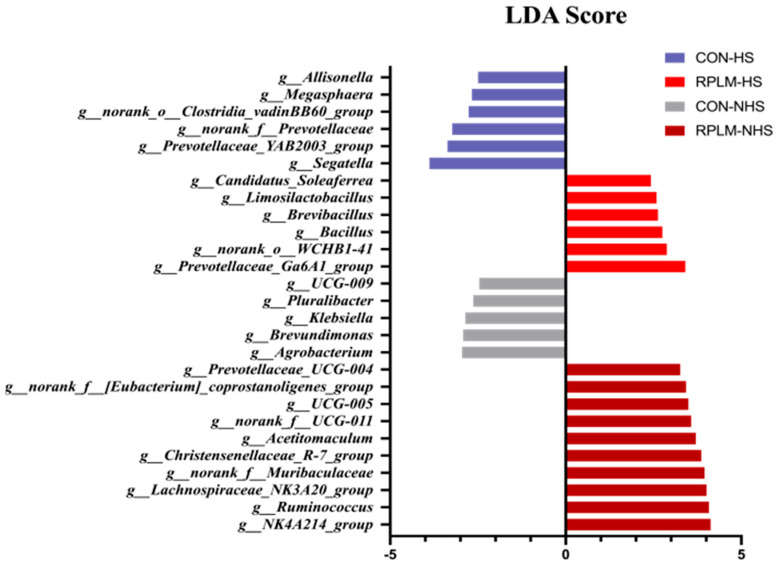
The bar chart displays the LDA values of distinct microbial species, visually illustrating the magnitude of influence that characteristic species from different groups exert on the observed differences (LDA score threshold > 2.5, *p* < 0.05). The LDA discriminant analysis identifies microbial taxa with statistically significant effects across multiple groups. These LDA scores, derived from linear discriminant analysis (a form of linear regression), indicate that higher scores correspond to a greater contribution of species abundance to the observed differences between groups.

The differential microbiota that varied in response to RPM and RPL treatments as well as environmental conditions were further identified using linear discriminant analysis effect size (LEfSe bar chart) (Figure 9). Using the default LDA threshold value of ±2.5, a total of 6, 6, 5, and 10 differential taxa were detected in the CON-HS group, RPLM-HS group, CON-NHS group, and RPLM-NHS group, respectively. The bacterial biomarkers identified in the CON-HS group included *g__Segatella*, *g__Prevotellaceae_YAB2003_group*, and *g__norank_f__Prevotellaceae*, among others. In the RPLM-HS group, the key biomarkers were *g__Prevotellaceae_Ga6A1_group*, *g__norank_o__WCHB1-41*, *g__Bacillus* and etc. For the CON-NHS group, the biomarkers consisted of *g__Agrobacterium*, *g__Brevundimonas*, *g__Klebsiella,* etc. Finally, in the RPLM-NHS group, the dominant bacterial biomarkers included *g__NK4A214_group*, *g__Ruminococcus*, among others.

**Figure 10:**
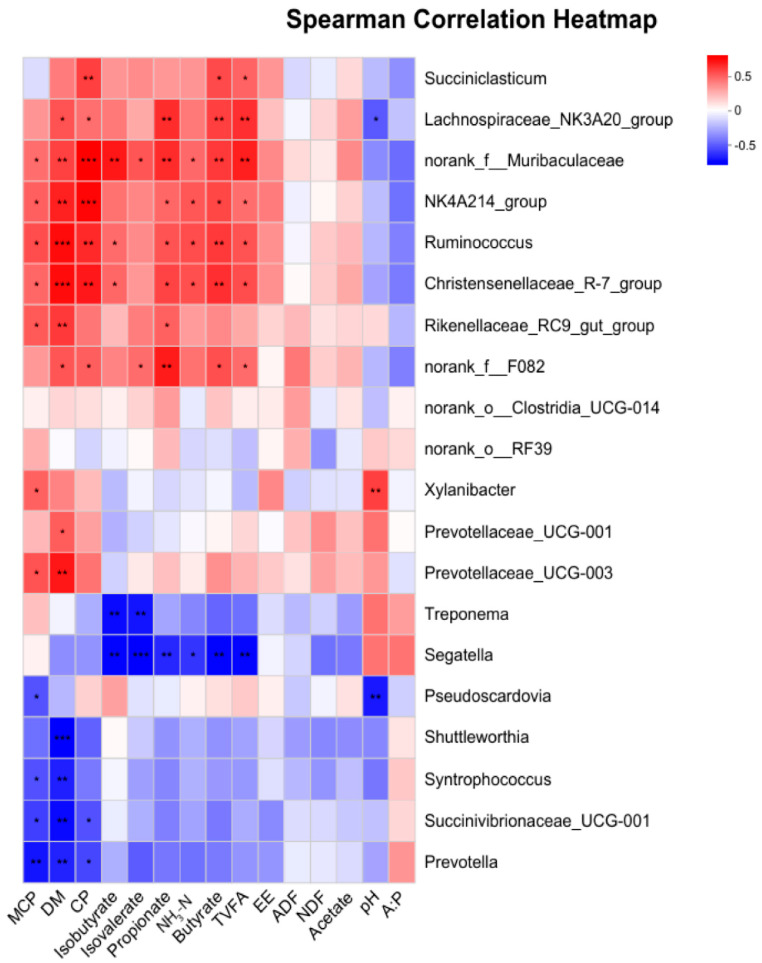
The correlation heatmap illustrates the relationships between microbial species and environmental factors, providing a visual representation of both the strength and significance of correlations across multiple environmental variables and different microbial taxa. The *X*-axis represents environmental factors, while the *Y*-axis corresponds to microbial genera. Correlation coefficients (R values) and statistical significance (*p* values) were calculated for each pairwise comparison. R values are represented using a color gradient, where positive and negative correlations are indicated by distinct hues. Significant associations are annotated with asterisks: * for 0.01 < *p* ≤ 0.05, ** for 0.001 < *p* ≤ 0.01, *** for *p* ≤ 0.001.

A correlation analysis was performed to evaluate the relationships between apparent digestibility, rumen fermentation parameters, and bacterial genera in the CON and RPLM groups (Figure 10). The results demonstrate that the relative abundances of bacterial genera—including *norank_f__Muribaculaceae*, *NK4A214_group*, *Christensenellaceae_R-7_group*, and *Ruminococcus*—are significantly positively correlated with the apparent digestibility of DM and CP (*p* < 0.01), as well as with concentrations of MCP, propionate, butyrate, TVFA, and NH_3_-N (*p* < 0.05). Notably, *norank_f__F082* exhibits significant positive correlations with DM (r = 0.4713) and CP (r = 0.44966) digestibility, isovalerate (r = 0.41418), butyrate (r = 0.48837), and TVFA (r = 0.41652) (*p* < 0.05), and shows a highly significant positive correlation with propionate concentration (*p* < 0.01). In contrast, the relative abundances of *Succinivibrionaceae_UCG-001* (r = −0.59391), *Prevotella* (r = −0.53913), and *Syntrophococcus* (r = −0.5487) are significantly negatively correlated with DM digestibility (*p* < 0.01), whereas *Shuttleworthia* (r = −0.64522) displays an extremely significant negative correlation (*p* < 0.001). Furthermore, *Segatella* exhibits either significant or highly significant negative correlations across most rumen fermentation parameters.

## 4. Discussion

Apparent digestibility in dairy cows serves as an indicator of their efficiency in utilizing dietary nutrients. Extensive research has demonstrated that heat stress significantly reduces both feed intake and apparent digestibility in dairy cattle [31,32]. This study reveals that the RPLM group exhibited a highly significant improvement in the apparent digestibility of DM and CP compared to the CON group (*p* < 0.01). Moreover, a significant interaction effect between treatment and environmental conditions was observed for DM digestibility. The apparent digestibility of NDF also increased significantly in the RPLM group (*p* < 0.05). Studies have shown that under heat stress, apparent digestibility in dairy cows is often not significantly affected and may even increase in some cases. However, this phenomenon is primarily attributed to reduced feed intake and slowed gastrointestinal motility, which collectively delay the passage of digesta through the digestive tract [33,34]. Current evidence suggests that changes in apparent digestibility are often linked to alterations in the rumen microbial community induced by supplementation with rumen-protected amino acids such as RPM or RPL [3]. Although these amino acids are protected by coating layers designed to prevent ruminal degradation, partial breakdown may still occur in the rumen. The released amino acids can then be either deaminated to ammonia and subsequently assimilated by microorganisms, or directly taken up and metabolized by rumen microbes to support their growth. However, several studies have reported that supplementation with either RPL or RPM alone does not significantly affect apparent digestibility in dairy cows [35,36,37]. A potential explanation for the observed differences in this study is the concurrent administration of RPL and RPM. The addition of RPL may enhance the intestinal absorption and metabolic efficiency of RPM while also influencing rumen microbial dynamics. By bypassing ruminal degradation, RPL and RPM reduce competition between rumen microorganisms and the host for these essential amino acids, thereby potentially improving fiber and dry matter digestibility. Furthermore, elevated ambient temperatures have been shown to inhibit the activity of digestive enzymes in the gastrointestinal tract, which may compromise nutrient digestion and absorption [38,39]. In this experiment, the RPLM group exhibited a significant increase in dry matter digestibility, along with a significant interaction effect between environmental conditions and treatment. These findings further confirm that heat stress negatively affects DM digestibility in dairy cows, and supplementation with RPL and RPM can mitigate this adverse impact. Additionally, RPL and RPM modulate the rumen microbial community, optimize amino acid balance, and enhance ruminal fermentation efficiency and dry matter digestibility. Therefore, we speculate that simultaneous supplementation of RPL and RPM may improve nutrient utilization efficiency in dairy cows through stabilization of the rumen microbial population and enhancement of intestinal health. However, discrepancies between this study and previous reports may arise from variations in supplementation levels, differences in dietary formulations, or inherent individual variability among dairy cows.

Previous in vitro studies have demonstrated that RPL and RPM can influence rumen fermentation parameters and microbial composition [40,41]. Our in vivo experiments further revealed that NH_3_-N concentration in the RPLM group exhibited a significant treatment effect, along with a highly significant environmental effect, despite the absence of significant differences in MCP between groups. These findings suggest that supplementation with RPM and RPL enhances nitrogen utilization efficiency in the rumen of dairy cows, optimizes the profile of metabolic amino acids, and promotes the synthesis of microbial protein, which can subsequently be absorbed and utilized in the small intestine. Consistent with our findings, previous studies have reported that supplementation with RPM or RPL significantly increases ruminal MCP content while reducing NH_3_-N levels to varying degrees [37,42]. Furthermore, as previously observed, RPM and RPL significantly improve the apparent digestibility of DM, NDF, and ADF, thereby synchronizing the supply of energy and nitrogen to ruminal microorganisms. This synchronization is likely to enhance MCP synthesis efficiency and reduce ammonia accumulation caused by insufficient energy availability [42]. The amino acids released in the rumen can be directly assimilated by ruminal microorganisms, serving as substrates for MCP synthesis or energy production, thereby mitigating excessive proteolysis and deamination processes and ultimately contributing to the regulation of NH_3_-N accumulation [43,44]. Additionally, our results showed that acetate, propionate, butyrate, and TVFA were all significantly influenced by treatment (*p* < 0.01). Moreover, butyrate and isovalerate exhibited highly significant environmental effects (*p* < 0.01), indicating that environmental conditions play a crucial role in shaping ruminal fermentation profiles.

Studies have demonstrated that elevated ambient temperatures can decrease DMI in dairy cows by 20% to 40%, thereby reducing the quantity of fermentable organic matter entering the rumen. This limitation in substrate availability directly constrains the synthesis of VFAs [45]. Furthermore, HS has been shown to alter the composition of the ruminal microbiota, particularly by reducing the relative abundance of fiber-degrading bacterial populations, which in turn compromises fiber fermentation and subsequent acid production [46]. These factors may account for the highly significant environmental effects observed for butyrate and isovalerate in our study (*p* < 0.05). Following partial ruminal degradation of RPM and RPL, research indicates that acetate, butyrate and NH_3_-N are the primary end products of lysine metabolism [47]. Upon entering the rumen, RPM undergoes microbial conversion to 2-oxobutyric acid [47], which may subsequently be metabolized into propionate, along with minor quantities of acetate and butyrate [48]. This metabolic pathway could explain the significant treatment effects observed for acetate, propionate, and butyrate in the RPLM group (*p* < 0.05), as well as the significant treatment effect on NH_3_-N concentration, which was unexpectedly higher during non-heat stress periods. Collectively, these findings suggest that the observed alterations in ruminal fermentation profiles are likely attributable to the influence of RPL and RPM on the structure and functional capacity of the ruminal microbial community.

In our study, *Bacteroidota*, *Bacillota*, and *Pseudomonadota* were identified as the predominant phyla across all four experimental groups, a finding consistent with previous studies on ruminants [49,50]. Notably, *Bacillota* exhibited significant variation among the groups. Accumulating evidence suggests that both *Bacillota* and Bacteroidetes are closely associated with short-chain fatty acid (SCFA) metabolism. Specifically, *Bacillota* are primarily involved in the synthesis of butyrate and propionate, whereas Bacteroidetes mainly contribute to propionate production [51]. At the genus level, several taxa belonging to the *Bacteroidetes*, including *norank_f_Muribaculaceae* and *norank_f__F082*, showed significant differences in relative abundance. In contrast, *Christensenellaceae_R-7_group*, *Acetitomaculum*, and *norank_f__UCG-011* were classified under the *Bacillota*. Members of *Bacteroidetes* (and *Bacillota*) are known to produce α-amylase, α-1,2-mannosidase, and endo-1,4-β-mannosidase [52], and are more capable of degrading starch and other complex polysaccharides [53]. These findings align with the significant treatment effects observed in the RPLM group on propionate and butyrate concentrations. Correlation analysis further revealed that *norank_f_Muribaculaceae*, *norank_f__F082*, *Christensenellaceae_R-7_group*, and *NK4A214_group* exhibited significant or highly significant positive correlations with the concentrations of propionate, butyrate, isovalerate, and TVFA. The addition of RPL and RPM may provide additional nitrogen sources and fermentation substrates, thereby enhancing microbial metabolic activity and increasing SCFA production. The observed decrease in the relative abundance of *Segatella*, a member of the Bacteroidetes, may reflect alterations in rumen microbial community structure and substrate availability following RPL and RPM supplementation. The genus *norank_f__F082*, which exhibited significant differences under varying environmental conditions, showed a strong positive correlation with apparent digestibility of DM and CP, as well as with concentrations of propionate, butyrate, isovalerate, and TVFA. These results suggest that RPL and RPM supply slow-release amino acids, optimize microbial nitrogen utilization, and enhance *norank_f__F082*’s metabolic activity on fibrous substrates. Concurrently, the increased relative abundance of *Bacillota* in the RPLM group may strengthen the synergistic interactions between *Bacillota* and *Bacteroidetes*, thereby improving DM degradation. *NK4A214_group*, a member of the *Bacillota*, also showed significant differences in relative abundance. Functional genomic analysis of *NK4A214_group* revealed significant positive associations with metabolic pathways such as glycolysis, L-glutamate degradation, quinone/quinoxaline synthesis, and purine nucleotide degradation [54], indicating a strong capacity for degrading structural carbohydrates and producing VFAs. Similarly, *norank_f_Muribaculaceae* exhibited a highly significant positive correlation with DM and CP digestibility, as well as most rumen fermentation parameters. Metagenomic studies have shown that *Muribaculaceae* can ferment plant polysaccharides to produce acetate, propionate, and succinate, thereby supplying additional ATP for microbial protein synthesis in the rumen [55]. The addition of RPL and RPM may further enhance this energy-nitrogen synergistic effect, reduce ammonia nitrogen losses, and improve the apparent digestibility of DM and CP. It has been reported that supplementation with RPM and RPL promotes the enrichment of *Akkermansia muciniphila* in the colon [56], a genus belonging to the phylum *Verrucomicrobia*—which exhibited significant differences in relative abundance in our study. This observation suggests that RPL and RPM supplementation may similarly influence the ruminal abundance of this taxon, potentially contributing to improved metabolic health and intestinal barrier function [57]. However, due to its extremely low relative abundance in the rumen, *Akkermansia muciniphila* was not detected as a dominant or differentially abundant genus in our analysis. LEFSe analysis further confirmed that *NK4A214_group*, *norank_f_Muribaculaceae*, *Christensenellaceae_R-7_group*, and *Acetitomaculum* were among the most discriminative taxa in the RPLM-NHS group. These findings suggest that ruminal microorganisms can dynamically adapt to environmental changes and re-establish a microbial structure that is more beneficial to host physiology. HS altered the relative abundance of nearly all bacterial genera, a phenomenon that was consistently observed in our interaction analysis of differential bacterial genera with relative abundance exceeding 0.5%, and has been corroborated by previous studies [58,59]. Although *Thermodesulfobacteriota* is not a dominant phylum in the rumen, it possesses sulfate-reducing and hydrogen-utilizing capabilities, potentially contributing to methane mitigation, sulfur cycling, and improved fiber fermentation efficiency [60]. Its activity may be particularly enhanced under high-sulfur dietary conditions.

During the experiment, our focus was strictly limited to aspects related to nutrient digestion and rumen fermentation, with no assessment of dairy cows’ production performance or serum biochemical indicators under varying environmental conditions. Given that RPAA may improve production performance—such as milk yield and milk protein content—by enhancing the supply of absorbable amino acids in the small intestine, as well as contribute to overall metabolic health, future studies will concurrently measure DMI, milk yield, and milk composition following supplementation with RPL and RPM across diverse environments. This approach aims to provide a comprehensive evaluation of the overall efficacy of RPAA in dairy cattle. Furthermore, expanding the sample size will help minimize confounding effects arising from individual animal variability on ruminal microbial composition.

## 5. Conclusions

This study demonstrated that dietary supplementation with 60 g/d RPL and 30 g/d RPM improved apparent nutrient digestibility in dairy cows under both heat-stress and non-heat-stress conditions, effectively mitigating the digestive inhibition associated with heat stress. Furthermore, the interaction between RPL and RPM supplementation and environmental conditions was examined, revealing that rumen microorganisms—particularly butyrate-producing bacteria—are influenced not only by environmental fluctuations but also by the main effect of RPAA supplementation. This dual influence contributed to enhanced rumen fermentation profiles during heat stress, as evidenced by increased production of propionate and butyrate. Future studies employing omics approaches, such as metagenomics, could further elucidate the expression of key genes involved in fiber degradation and butyrate synthesis pathways, thereby validating the functional mechanisms underlying microbial community improvements.

## Figures and Tables

**Table 1:** Feed ingredients and chemical composition (% of DM unless noted) of the experimental diets.

Items ^4^	CON	RPLM
Ingredient(%DM)		
Steam-flaked corn	10.42	10.42
Sugar beet pulp	4.74	4.74
Corn silage	28.97	28.97
Alfalfa hay	13.30	13.30
Cottonseed meal	6.33	6.33
Oat grass	2.23	2.23
BSG	4.89	4.89
Premixed supplement ^1^	29.12	29.12
Total	100	100
Chemical composition ^2,3^		
DM,% as fed	48.3	48.3
CP	15.27	15.27
EE	5.40	5.40
NDF	29.39	29.39
ADF	16.97	16.97
Starch	27.28	27.28
Met(g)	57.4	75.6
Met/MP	1.80	2.35
Lys(g)	202.1	225.4
Lys/MP	6.34	7.01
Lys:Met ratio	3.52	2.98
NEL(Mcal/kg) ^2^	1.74	1.74

(^1^) One kg premix contained the following: VA 400,000 IU, VD60000 IU Fe 400 mg, Cu 250 mg, Zn 3000 mg, Mn 350 mg, Se 15 mg, Co 35 mg. (^2^) Estimated using the NRC [1]) model based on DM intakes. (^3^) The nutrient levels are measured values. (^4^) CON = the CON group; RPLM = the RPLM group.

## Data Availability

Upon reasonable request, the datasets of this study can be available from the corresponding author.
